# Nephrotic Syndrome Due to Sunitinib: A Rare Complication of Treatment of Renal Cell Carcinoma

**DOI:** 10.7759/cureus.56178

**Published:** 2024-03-14

**Authors:** Sunny Malde, Pranjal Kashiv, Sushrut Gupta, Shubham Dubey, Manish Balwani, Amit Pasari, Kapil N Sejpal, Prasad Gurjar

**Affiliations:** 1 Nephrology, Jawaharlal Nehru Medical College, Wardha, IND; 2 Nephrology, Saraswati Kidney Care Center, Nagpur, IND

**Keywords:** hypertension, hypothyroidism, targeted therapy, renal cell carcinoma, nephrotic syndrome, sunitinib

## Abstract

This case report details a 62-year-old male with a history of right renal cell carcinoma (RCC) who developed sunitinib-induced nephrotic syndrome during treatment. The patient had a complex medical history, including a right nephrectomy in 2009, brain metastasis excisions in 2011 and 2012, and prolonged sunitinib therapy. Hypothyroidism, hypertension, and various surgeries further complicated his clinical picture. In April 2022, the patient presented with bilateral pedal edema, acute kidney injury superimposed on chronic kidney disease, and proteinuria. Upon examination, the decision was made to discontinue sunitinib, leading to the resolution of nephrotic syndrome. Adjustments in thyroxine dosage were made, and pharmacological interventions were employed to manage proteinuria and renal dysfunction. A multidisciplinary approach involving oncologists, nephrologists, and endocrinologists was essential in achieving a favorable outcome. The case highlights the intricate balance required in managing patients undergoing targeted cancer therapies, emphasizing the importance of vigilant monitoring, prompt intervention, and a collaborative approach for optimal patient care.

## Introduction

Renal cell carcinoma (RCC) represents a substantial portion of urological malignancies, with clear cell carcinoma emerging as the predominant histological subtype. The landscape of advanced RCC treatment has undergone significant transformation with the advent of targeted therapies like sunitinib, which offer improved rates of overall response and progression-free survival [1]. Sunitinib, classified as a tyrosine kinase inhibitor, exerts its anti-tumor effects by targeting multiple receptor tyrosine kinases implicated in angiogenesis and tumor cell proliferation. Despite its demonstrated efficacy in RCC, its utilization is associated with a spectrum of adverse effects, including hypertension, hypothyroidism, and renal dysfunction [2,3].

The renal complications stemming from sunitinib are increasingly acknowledged, spanning from proteinuria to nephrotic syndrome. While proteinuria is a well-documented side effect observed in a significant number of patients, nephrotic syndrome remains relatively uncommon [4]. Nephrotic syndrome, characterized by proteinuria exceeding 3.5 g/day, hypoalbuminemia, and edema, presents notable challenges in managing patients undergoing sunitinib therapy [5]. The precise pathophysiological mechanisms underlying sunitinib-induced nephrotic syndrome necessitate further elucidation. It is postulated that the drug may precipitate podocyte injury, thereby enhancing glomerular permeability and subsequently leading to proteinuria [6]. Additionally, pre-existing conditions such as hypertension and chronic kidney disease may exacerbate the development and severity of renal complications [7].

Given the potential impact on overall prognosis and quality of life, early recognition and management of sunitinib-induced nephrotic syndrome are imperative. A multidisciplinary approach involving oncologists, nephrologists, and endocrinologists is essential for comprehensive patient care [8]. This case report contributes to the expanding body of evidence on the renal complications associated with sunitinib, underscoring the necessity for vigilant monitoring, timely intervention, and a tailored approach to mitigate adverse effects while optimizing oncological outcomes in patients with advanced RCC.

## Case presentation

A 62-year-old male presented to the outpatient department of a tertiary care hospital with complaints of bilateral foot swelling persisting for one month. Upon reviewing his medical history, the patient disclosed a prior admission to a specialty hospital 14 years ago, where he received a diagnosis of right RCC. Subsequently, he underwent a right-sided nephrectomy in 2009. In 2011, the patient was diagnosed with brain metastasis, necessitating surgical excision in both 2011 and 2012. Post-surgery in 2011, he commenced sunitinib therapy, which has been ongoing to date. Routine investigations in 2011 revealed hypothyroidism, with a serum TSH level of 23.09 mIU/L, prompting the initiation of oral thyroxine (50 μg).

The patient remained under regular follow-up until 2015 when he was diagnosed with renal dysfunction, categorized as chronic kidney disease. However, he was lost to follow-up between June 2015 and 2021, during which time he continued taking sunitinib without medical supervision. In April 2022, he presented to the outpatient department with complaints of progressively increasing pitting edema and abdominal distension. On examination, his blood pressure was elevated at 160/100 mmHg, and he exhibited generalized swelling. Urine analysis revealed significant albuminuria (3+) without red blood cells. Routine investigations showed elevated serum creatinine (2.37 mg/dL), total cholesterol (320 mg/dL), and decreased serum albumin (2.5 g/dL), along with an elevated urine protein-to-creatinine ratio (UPCR) of 3.6 and uric acid level of 7.7 mg/dL. He was diagnosed with hypertension but was not receiving any medication for it.

**Figure 1:**
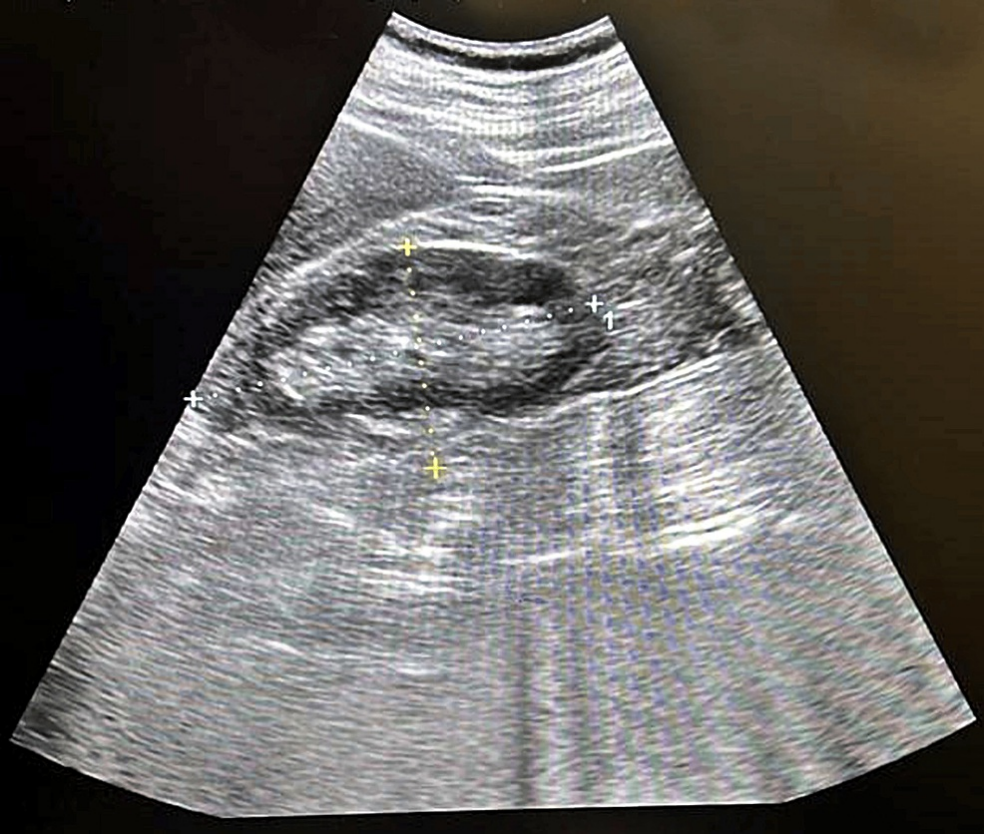
Shows left kidney displayed increased cortical echogenicity with maintained corticomedullary differentiation, measuring 10×4.6 cm

Abdominal ultrasonography demonstrated the post-nephrectomy status of the right kidney, while the left kidney displayed increased cortical echogenicity with maintained corticomedullary differentiation, measuring 10x4.6 cm (Figure 1). Renal Doppler studies excluded renal artery stenosis, and anti-nuclear antibody (ANA) testing by immunofluorescence returned negative. Based on the collective findings, a diagnosis of nephrotic syndrome was established, prompting the discontinuation of sunitinib.

Following the cessation of sunitinib, the thyroxine dosage was increased to 75 μg once daily. Within 15 days of halting sunitinib, pedal edema began to diminish and eventually resolved completely. Ongoing management included treatment for hypertension (amlodipine: 5 mg and moxonidine: 0.2 mg), hypothyroidism (thyroxine: 75 μg), and aspirin. A tentative plan was outlined to cautiously reintroduce sunitinib at half dosage if needed after the resolution of proteinuria. However, if nephrotic range proteinuria recurred, permanent discontinuation of sunitinib would be considered, contingent upon consultation with a medical oncologist.

## Discussion

The presented case underscores the complex interplay between targeted cancer therapies, such as sunitinib, and their potential adverse effects on renal function. Sunitinib, a multi-targeted tyrosine kinase inhibitor, is commonly used in the treatment of advanced RCC. However, its association with renal toxicity, including nephrotic syndrome, has been documented in the literature [2,9]. Nephrotic syndrome is a rare but recognized complication of sunitinib therapy, encompassing hypoalbuminemia, proteinuria, edema, and hyperlipidemia [9,10]. In this case, the patient developed nephrotic syndrome after prolonged exposure to sunitinib. The decision to discontinue sunitinib was crucial in the management of this case, leading to a subsequent improvement in renal function and resolution of proteinuria.

The patient's history of hypertension and chronic kidney disease likely contributed to the development of sunitinib-induced nephrotic syndrome. The chronic renal impairment, compounded by the vasoconstrictive effects of sunitinib, may have further exacerbated the renal dysfunction [11]. It is noteworthy that the patient's hypertension required a combination of antihypertensive medications, emphasizing the importance of blood pressure control in individuals undergoing sunitinib therapy. The management of sunitinib-induced nephrotic syndrome necessitated a multidisciplinary approach involving oncologists, nephrologists, and endocrinologists. Close collaboration among these specialties is vital for optimizing patient outcomes and addressing the intricate medical issues associated with targeted cancer therapies.

The patient's history of hypothyroidism adds another layer of complexity to the case. Hypothyroidism is considered a potential side effect of sunitinib [12]. In this case, the adjustment of thyroxine dosage after discontinuation of sunitinib highlights the need for vigilant monitoring of thyroid function in patients receiving this tyrosine kinase inhibitor. The decision to potentially reintroduce sunitinib at a reduced dose, contingent upon the resolution of proteinuria, reflects a careful risk-benefit assessment. Monitoring for the recurrence of nephrotic range proteinuria is crucial, and any decision to permanently discontinue sunitinib will be made in consultation with a medical oncologist.

## Conclusions

This case underscores the significance of recognizing and managing sunitinib-induced nephrotic syndrome in a patient with a history of RCC. A multidisciplinary approach was pivotal in discontinuing sunitinib, adjusting thyroid medication, and implementing pharmacological interventions. The patient's positive response to treatment highlights the importance of vigilance in monitoring and a collaborative decision-making process for the potential re-initiation of sunitinib. This case underscores the need for heightened awareness of the nephrotoxic effects of targeted cancer therapies, guiding optimal patient care. Ongoing research will further refine our understanding of these adverse effects, aiding in tailored treatment decisions.
